# Nilotinib-Associated Destructive Thyroiditis

**DOI:** 10.1155/2015/736092

**Published:** 2015-05-07

**Authors:** Suhalia Bakerywala, Monica D. Schwarcz, Michael D. Goldberg, Guy Valiquette, Irene A. Weiss

**Affiliations:** Division of Endocrinology, Department of Medicine, Westchester Medical Center, New York Medical College, Valhalla, NY 10595, USA

## Abstract

Protein tyrosine kinase inhibitors are currently an important drug class in the treatment of leukemia. They represent targeted cancer therapy and have become the treatment of choice in chronic myeloid leukemia. Tyrosine kinases are enzymes expressed in multiple tissues and are involved in several signaling pathways influencing cellular growth. Below we describe a patient who developed an unusual complication of tyrosine kinase inhibitor therapy: thyrotoxicosis due to destructive thyroiditis. We review the pathophysiology of tyrosine kinase inhibitor-induced thyroid dysfunction particularly with regard to new second-generation tyrosine kinase inhibitors.

## 1. Introduction

Nilotinib is a second-generation tyrosine kinase inhibitor approved for the treatment of adults with Philadelphia chromosome-positive chronic myeloid leukemia (Ph+ CML) [1, 2]. Tyrosine kinase inhibitors (TKIs) as a class compete with the ATP binding site of oncogenic tyrosine kinases leading to blocking of pathways essential for tumor cell survival. TKIs, particularly 1st generation formulations, are associated quite commonly with thyroid dysfunction particularly hypothyroidism. Second-generation TKIs appear to have fewer side effects related to thyroid dysfunction, and if thyroid changes do occur, they are usually transient [3]. Below we discuss an unusual case of transient thyrotoxicosis related to nilotinib therapy.

## 2. Case Presentation

A 53-year-old female with chronic myeloid leukemia (CML) presented to our emergency department with one week of severe palpitations, tremors, and anxiety. She also complained of a rapidly enlarging painful neck mass and difficulty swallowing. The symptoms began two weeks after the initiation of nilotinib therapy. There was no history of any recent upper respiratory tract infections, fevers, or viral syndromes. She had no personal or family history of any thyroid disorders.

On physical examination, the patient was afebrile and slightly agitated. She was tachycardic, with a heart rate ranging from 120 to 150 beats per minute and an irregularly irregular heart rhythm. An electrocardiogram confirmed atrial fibrillation. The thyroid gland was symmetrically enlarged to approximately 60 grams, was firm, and was diffusely tender to palpation. A fine tremor of the outstretched hands and brisk deep tendon reflexes were noted.

Initial laboratory work-up revealed a suppressed thyroid-stimulating hormone (TSH) with elevated total T3 and free T4. Thyroglobulin was markedly elevated, and the titer of anti-thyroperoxidase antibody was strongly positive. Thyroid-stimulating immunoglobulin was negative (see Table 1).

The erythrocyte sedimentation rate was elevated at 80 mm/hr (1–25 mm/h). Anemia and thrombocytopenia were present, consistent with the history of CML. Serum electrolytes, renal function and liver function tests were all unremarkable.

The patient was admitted to the intensive care unit, nilotinib was discontinued, and stress doses of hydrocortisone were administered. The atrial fibrillation was rate-controlled with propranolol infusion.

**Figure 1:**
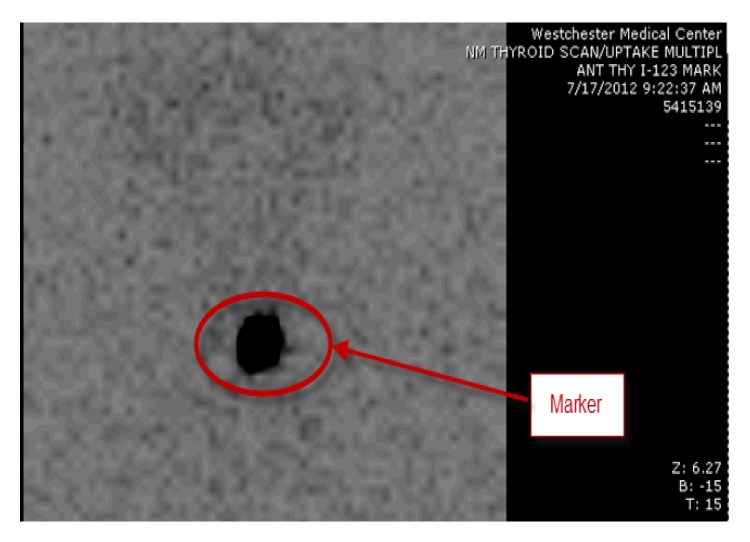
Radioactive iodine uptake at 24 hours with very low uptake in thyroid bed.

The 24-hour radioactive iodine uptake was 1.7% (normal, 15–35%), and no thyroid tissue was visualized on scan (see Figure 1).

Over the next five days, with continuing high dose hydrocortisone, total T3 level decreased from 229.6 to 67.2 ng/dL, and the free T4 level decreased from 4.6 to 2.6 ng/dL; the TSH level remained suppressed (see Table 1). Thyromegaly decreased rapidly and her neck pain was significantly reduced over a period of two to four days. Heart rhythm reverted back to a normal sinus rhythm. On hospital day six, the patient was discharged home, with instructions to taper oral steroids over the following two weeks. She has followed subsequently with an outside physician and is now euthyroid.

## 3. Discussion

Tyrosine kinase inhibitors block the BCR-ABL gene product responsible for symptoms of CML. Tyrosine kinases are enzymes that catalyze the transfer of phosphate from ATP to tyrosine residues in polypeptides [4]. These enzymes are widely distributed throughout multiple tissue types and are involved in signaling pathways that regulate key cellular functions including proliferation, differentiation, growth, and apoptosis. More than 70% of known oncogenes and protooncogenes encode for tyrosine kinases [5], and receptor tyrosine kinases are known to be overexpressed in malignant diseases. Small molecule tyrosine kinase inhibitors (TKIs) have been designed to disrupt the catalytic activity of tyrosine kinases, thus disrupting the growth of malignant cells. As tyrosine kinases are expressed ubiquitously, TKIs have been noted to have several “off-target” effects. Thyroid dysfunction appears to be one of the off-target effects of TKIs. Although TKIs were initially thought of as targeted therapy, normal healthy tissue may also be affected as tyrosine kinases are ubiquitously expressed, including in thyroid tissue [6, 7].

Kim and colleagues retrospectively assessed the effect of nilotinib on thyroid function in 55 patients with Ph+ CML. Of the 30 patients without preexisting thyroid dysfunction, 18 patients (60%) developed a suppressed TSH, and 12 patients (40%) developed an elevated TSH. Four patients (of whom 3 had positive anti-thyroid antibodies) had evidence of thyroiditis, with an episode of transient thyrotoxicosis followed by hypothyroidism [8].

Here we present another case of new-onset thyroid dysfunction in a patient who had recently begun therapy with nilotinib. We postulated that the thyrotoxicosis was due to drug-induced thyroiditis. This was based on the presence of a diffusely enlarged and painful thyroid gland with elevated ESR and low radioiodine uptake. Nilotinib was discontinued, and the patient was treated with steroids and beta-blockers, with rapid resolution of thyrotoxicosis.

Understanding the etiology of TKI-induced thyroid dysfunction is a topic of considerable interest. Torino and colleagues in a 2009 review [9] suggested several potential mechanisms in which both thyroid hormone syntheses may be impaired and a destructive thyroiditis may develop later in the course of therapy. First, TKIs cause apoptosis of the thyroid follicular cells, leading to destructive thyroiditis. Secondly, prevention of vascular endothelial growth factor (VEGF) binding to normal thyroid cells and/or inhibiting thyroid blood flow may cause thyroiditis and thyroid dysfunction. Thirdly, inhibition of thyroid peroxidase activity may result in decreased thyroid hormone synthesis. Partial inhibition of TKI of pituitary or hypothalamic thyroid hormone transporter (TH) feedback, may alter the metabolism of TH and contribute to TKI-mediated impairments of thyroid function. Lastly, a yet to be described autoimmune mechanism affecting thyroid function may result in hypo- or hyperthyroidism.

TKIs are now used in the treatment of selected patients with advanced thyroid cancer. Multiple receptor tyrosine kinases are overexpressed in thyroid cancer, including RET, rapidly accelerated fibrosarcoma (RAF) kinases, VEGF, mesenchymal epithelial transition factor, and epidermal growth factor. Because multiple receptor tyrosine kinases are overexpressed in thyroid cancer, TKIs may also represent new strategies in treating advanced thyroid cancers via targeting signaling by VEGF and its receptors. Various TKIs are currently being studied for their potential use in treatment of advanced and metastatic thyroid cancer. Vandetanib, which inhibits VEGF receptor dependent tumor angiogenesis and epidermal growth factor receptor (EGFR-) and RET-mediated tumor cell proliferation, along with cabozantinib, is now FDA approved for treatment of medullary thyroid cancer [10]. Additionally, sorafenib and lenvatinib have been recently FDA approved for use in patients with advanced and refractory differentiated thyroid cancer [11].

## 4. Conclusion

Prospective trials are needed to accurately define the incidence of thyroid dysfunction during therapy with many newly approved tyrosine kinase inhibitors used to treat various malignancies. Initial screening and periodic surveillance of thyroid function during tyrosine kinase inhibitor therapy are prudent given the frequency of thyroid dysfunction with these drugs. In addition, because multiple receptor tyrosine kinases are overexpressed in thyroid cancer, TKIs represent promising new strategies for the treatment of advanced thyroid cancers; in particular, TKIs have been shown to have potential in differentiated thyroid carcinoma [12, 13].

## Figures and Tables

**Table 1:** Thyroid laboratory results during hospital admission.

Thyroid function values	Admission day					
	Day 1	Day 2	Day 3	Day 4	Day 5	Day 6
TSH (0.35–4.7) mIU/L	0.003	<0.002	0.003		<0.002	
T4 total (4.5–12) *μ*g/dL			17.1			
Free T4 (0.7–1.9) ng/dL	4.6	3.6	3.7		2.8	2.6
T3 total (79–149) ng/dL	229.6		116.5	83.8	67.2	
